# Poly[ethano­lbis(μ_3_-2-thio­xo-1,2-dihydro­pyridin-1-olato)dilithium(I)]

**DOI:** 10.1107/S1600536808003619

**Published:** 2008-02-08

**Authors:** Jens Hartung, Nina Schneiders, Ingrid Svoboda, Hartmut Fuess

**Affiliations:** a Fachbereich Chemie, Organische Chemie, Technische Universität Kaiserslautern, Erwin-Schrödinger-Strasse, D-67663 Kaiserslautern, Germany; b Strukturforschung, FB 11, Material- und Geowissenschaften, Technische Universität Darmstadt, Petersenstrasse 23, D-64287 Darmstadt, Germany

## Abstract

The title compound, [Li_2_(C_5_H_4_NOS)_2_(C_2_H_6_O)]_*n*_, having two formula units in the asymmetric unit, forms infinite chains of Li_2_O_2_ rhombi along *b*, consisting of four independent Li and O atoms. Metal binding to 2-thio­oxo-1,2-dihydro­pyridin-1-olate occurs in a bidentate fashion *via* O and S, and in a monodentate manner *via* the N-oxide O atom. π–π Inter­actions between polymeric chains are evident from centroid-to-centroid distances of pyridine­thione fragments of 3.461 (6)–3.607 (6) Å. The N—O and C—S bond lengths are distinctively different from those in hitherto investigated Ni^II^, Zn^II^ and (H_3_C)_2_Tl^III^ complexes of 2-thio­oxo-1,2-dihydro­pyridin-1-olate, but correlate with those reported for 1-hydr­oxy- and 1-alkoxy­pyridine-2(1*H*)-thio­nes in the solid state.

## Related literature

For related literature, see: Barnett *et al.* (1977 ▶); Castaño *et al.* (1988 ▶); Chen *et al.* (1991 ▶); Hartung, Hiller *et al.* (1996 ▶); Hartung, Svoboda *et al.* (1996 ▶); Hartung *et al.* (1999 ▶, 2007 ▶).
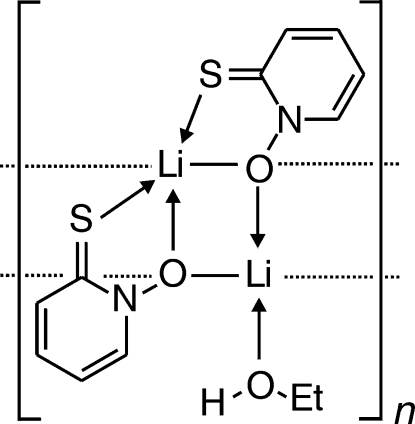


## Experimental

### 

#### Crystal data

[Li_2_(C_5_H_4_NOS)_2_(C_2_H_6_O)]
*M* *_r_* = 312.25
Monoclinic, 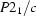

*a* = 22.492 (7) Å
*b* = 7.047 (2) Å
*c* = 20.881 (7) Å
β = 119.31 (4)°
*V* = 2886.0 (19) Å^3^
*Z* = 8
Mo *K*α radiation
μ = 0.38 mm^−1^
*T* = 300 (2) K
0.50 × 0.12 × 0.08 mm

#### Data collection

Oxford Diffraction Xcalibur diffractometer with a Sapphire CCD detector
Absorption correction: multi-scan [*CrysAlis RED* (Oxford Diffraction , 2006 ▶); analytical numeric absorption correction using a multifaceted crystal model based on expressions derived by Clark & Reid (1995 ▶)] *T*_min_ = 0.835, *T*_max_ = 0.971
16998 measured reflections
5597 independent reflections
1539 reflections with *I* > 2σ(*I*)
*R*_int_ = 0.086

#### Refinement

*R*[*F* ^2^ > 2σ(*F* ^2^)] = 0.045
*wR*(*F* ^2^) = 0.100
*S* = 0.71
5597 reflections
383 parameters
H-atom parameters constrained
Δρ_max_ = 0.44 e Å^−3^
Δρ_min_ = −0.32 e Å^−3^

### 

Data collection: *CrysAlis CCD* (Oxford Diffraction, 2006 ▶); cell refinement: *CrysAlis RED* (Oxford Diffraction, 2006 ▶); data reduction: *CrysAlis RED*; program(s) used to solve structure: *SHELXS97* (Sheldrick, 2008 ▶); program(s) used to refine structure: *SHELXL97* (Sheldrick, 2008 ▶); molecular graphics: *ORTEP-3* (Farrugia, 1997 ▶); software used to prepare material for publication: *SHELXL97*.

## Supplementary Material

Crystal structure: contains datablocks I, global. DOI: 10.1107/S1600536808003619/cv2377sup1.cif

Structure factors: contains datablocks I. DOI: 10.1107/S1600536808003619/cv2377Isup2.hkl

Additional supplementary materials:  crystallographic information; 3D view; checkCIF report

## Figures and Tables

**Table 1:** Selected interatomic distances (Å) *Cg*1, *Cg*2, *Cg*3 and *Cg*4 are the centroids of atoms N4,C16–C20, N1,C1–C5, N3,C11–C15 and N2,C6–C10, respectively.

*Cg*1⋯*Cg*2	3.470 (6)
*Cg*1⋯*Cg*2^i^	3.596 (6)
*Cg*3⋯*Cg*4	3.461 (6)
*Cg*3⋯*Cg*4^i^	3.607 (6)

Symmetry code: (i) 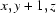.

**Table 2:** Hydrogen-bond geometry (Å, °)

*D*—H⋯*A*	*D*—H	H⋯*A*	*D*⋯*A*	*D*—H⋯*A*
O5—H5*A*⋯S1^i^	0.82	2.39	3.205 (3)	174
O6—H6*A*⋯S3^ii^	0.82	2.41	3.226 (4)	172

Symmetry codes: (i) 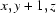; (ii) 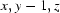.

**Fig. 1.**
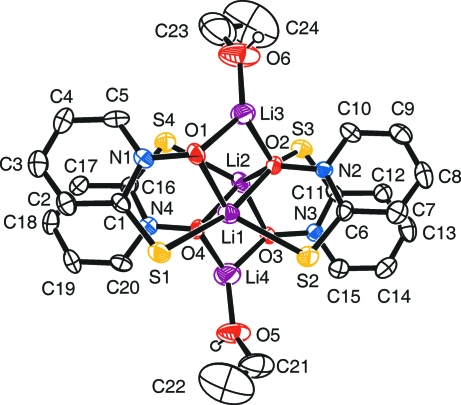
Molecular structure of (I). Displacement ellipsoids are plotted at the 50% probability level (O atoms are depicted in red, N in blue, C in yellow and Li in purple). C-bound H atoms omitted for clarity.

## supplementary crystallographic information

### Comment

2-Thiooxo-1,2-dihydropyridine-1-olate is an ambident nucleophile that is preferentially alkylated at sulfur in the presence of hard countercations, such as Na+ (Hartung *et al.*, 1999). The reactivity of the title compound, however, does not fit into this general scheme. Its inherent low reactivity toward strong electrophiles in association with a slight preference for the *O*-alkylation prompted us to explore its solid state geometry at 300 K. Diffraction experiments performed at 100 K and 150 K surprisingly did not afford data sets of an improved quality. The results of the structure investigation are summarized in the following section.

Formula 1, Figure 1

The title compound, (I), crystallizes in monoclinic space group *P*2_1_/*c* (*Z* = 4). Its structure is composed of infinite chains of Li_2_O_2_ rhombi along *y* consisting of four independent Li and O atoms (Figure 1). Neighbouring segments are tilted by approximately 90 ° in an accordion-like manner. π···π Interactions between polymeric chains are evident from centroid-to-centroid distances of pyridinethione fragments (Table 1). Metal binding to 2-thiooxo-1,2-dihydropyridine-1-olate occurs in two different ways. Li1 and Li2 are chelated *via* S and O to two molecules of 2-thiooxo-1,2-dihydropyridine-1-olate. In both instances, one of the ligands places the metal closer toward the heterocyclic plane [Li1—O2—N2—C6 = 13.6 (6)°, Li2—O4—N4—C16 = -15.0 (6)°] than in the other [Li1—S1—C1—N1 = -34.5 (4)°, Li2—S3—C11—N3 = 29.9 (4)°]. A fifth contact to Li1 and Li2 occurs *via* N-oxide binding of O2 and O4, giving rise to irregularly shaped polyhedra. Li3 and Li4 are located in distorted tetrahedral coordination polyedra that are composed of three N-oxide O-atoms [O1, O2, and O3 for Li3 and O1, O3, and O4 for Li4] and one O-atom from an ethanol solvate molecule [O5 for Li3 and O6 for Li4].

The observed parameters, in particular those of the central thiohydroxamate functionality of independent 2-thiooxo-1,2-dihydropyridine-1-olato entities in (I) [N1—O1 = 1.389 (5) Å, N2—O2 = 1.383 (4) Å, N3—O3 = 1.383 (4) Å, N4—O4 = 1.390 Å, C1—S1 = 1.654 (5) Å, C6—S2 = °1.671 (5), C11—S3 = 1.647 (5) Å, C16—S4 = 1.655 (5) Å] are distinctively different from those reported for the corresponding subunits 2-alkylsulfanyl pyridine-1-oxides [N—O = 1.308 Å, C—S = 1.739 (3) Å] (Hartung, Svoboda *et al.*, 1996), bis[2-thiooxo-1,2-dihydropyridine-1-olato]nickel [N—O = 1.343 (3) Å and 1.344 (4) Å, C—S = 1.710 (3) Å and 1.712 (3) Å] (Chen *et al.*, 1991, Hartung *et al.*, 2007), the corresponding Zn(II) compound [N—O = 1.34 (1) Å and 1.37 (1) Å, C—S = 1.716 (9) Å and 1.719 (9) Å] (Barnett *et al.*, 1977), and the derived dimethylthallium(III) complex [N—O = 1.338 (9) Å, C—S = 1.736 (9) Å] (Castaño *et al.*, 1988). A reasonable correlation, on the other hand, is seen with distances reported for 1-hydroxypyridine-2(1*H*)-thione [N—O = 1.367 (3) Å, C—S = 1.684 (2) Å] (Hartung *et al.*, 1999) and 1-[*trans*-(4-*tert*-butylcyclohexyl-1-oxy)]pyridine-2(1*H*)-thione [N—O = 1.384 (4) Å, C—S = 1.666 (4) Å] (Hartung, Hiller *et al.*, 1996). These findings point to a significant statistical weight of the thione formula for representing major structural aspects of the title compound (I) in the solid state. A hypothesis that is evident from the current results suggests that comparatively strong ligand to metal interactions occur in (2-thiooxo-1,2-dihydropyridine-1-olato)lithium(I). These attractions are likely to reduce its reactivity toward alkyl halides or tosylates even in strong donor solvents such as dimethyl sulfoxide or dimethyl formamide (Hartung *et al.*, 1999), thus providing an explanation of its unusual reactivity.

### Experimental

Crystals suitable for X-ray diffraction were grown by slow concentrating a saturated solution of (2-thiooxo-1,2-dihydropyridine-1-olato)lithium(I) (Hartung *et al.*, 1999) in EtOH at 293 K.

### Refinement

All H atoms were positioned geometrically (C—H = 0.93–0.96 Å, O—H = 0.82 Å), and treated as riding atoms, with U^iso^(H)=1.2 or 1.5 times U^eq^(parent atom).

### Crystal data

**Table:**

[Li_2_(C_5_H_4_NOS)_2_(C_2_H_6_O)]	*F*_000_ = 1296
*M**_r_* = 312.25	*D*_x_ = 1.437 Mg m^−^^3^
Monoclinic, *P*2_1_/*c*	Mo *K*α radiation λ = 0.71073 Å
Hall symbol: -P2ybc	Cell parameters from 1033 reflections
*a* = 22.492 (7) Å	θ = 2.2–20.6º
*b* = 7.047 (2) Å	µ = 0.38 mm^−^^1^
*c* = 20.881 (7) Å	*T* = 300 (2) K
β = 119.31 (4)º	Needle, colourless
*V* = 2886.0 (19) Å^3^	0.50 × 0.12 × 0.08 mm
*Z* = 8	

### Data collection

**Table:**

Oxford Diffraction Xcalibur diffractometer with a Sapphire CCD detector	5597 independent reflections
Radiation source: Enhance (Mo) X-ray Source	1539 reflections with *I* > 2σ(*I*)
Monochromator: graphite	*R*_int_ = 0.087
Detector resolution: 8.4012 pixels mm^-1^	θ_max_ = 26.4º
*T* = 300(2) K	θ_min_ = 4.1º
rotation method data acquisition using ω and φ scans	*h* = −28→27
Absorption correction: multi-scan[CrysAlis RED (Oxford Diffraction , 2006); analytical numeric absorption correction using a multifaceted crystal model based on expressions derived by Clark & Reid (1995)]	*k* = −8→4
*T*_min_ = 0.835, *T*_max_ = 0.971	*l* = −26→26
16998 measured reflections	

### Refinement

**Table:**

Refinement on *F*^2^	Secondary atom site location: difference Fourier map
Least-squares matrix: full	Hydrogen site location: inferred from neighbouring sites
*R*[*F*^2^ > 2σ(*F*^2^)] = 0.045	H-atom parameters constrained
*wR*(*F*^2^) = 0.100	*w* = 1/[σ^2^(*F*_o_^2^) + (0.039*P*)^2^] where *P* = (*F*_o_^2^ + 2*F*_c_^2^)/3
*S* = 0.71	(Δ/σ)_max_ = 0.073
5597 reflections	Δρ_max_ = 0.44 e Å^−^^3^
383 parameters	Δρ_min_ = −0.32 e Å^−^^3^
Primary atom site location: structure-invariant direct methods	Extinction correction: none

### Special details

**Table:**

Geometry. All e.s.d.'s (except the e.s.d. in the dihedral angle between two l.s. planes) are estimated using the full covariance matrix. The cell e.s.d.'s are taken into account individually in the estimation of e.s.d.'s in distances, angles and torsion angles; correlations between e.s.d.'s in cell parameters are only used when they are defined by crystal symmetry. An approximate (isotropic) treatment of cell e.s.d.'s is used for estimating e.s.d.'s involving l.s. planes.
Refinement. Refinement of *F*^2^ against ALL reflections. The weighted *R*-factor *wR* and goodness of fit *S* are based on *F*^2^, conventional *R*-factors *R* are based on *F*, with *F* set to zero for negative *F*^2^. The threshold expression of *F*^2^ > σ(*F*^2^) is used only for calculating *R*-factors(gt) *etc*. and is not relevant to the choice of reflections for refinement. *R*-factors based on *F*^2^ are statistically about twice as large as those based on *F*, and *R*- factors based on ALL data will be even larger.

### Fractional atomic coordinates and isotropic or equivalent isotropic displacement parameters (Å^2^)

**Table:**

	*x*	*y*	*z*	*U*_iso_*/*U*_eq_	
C1	0.3919 (2)	−0.1763 (6)	0.4763 (2)	0.0295 (12)	
C2	0.4254 (2)	−0.1700 (6)	0.4334 (3)	0.0386 (14)	
H2	0.4699	−0.1245	0.4558	0.046*	
C3	0.3978 (3)	−0.2239 (7)	0.3642 (3)	0.0458 (14)	
H3	0.4214	−0.2175	0.3381	0.055*	
C4	0.3336 (3)	−0.2888 (7)	0.3338 (3)	0.0447 (15)	
H4	0.3113	−0.3282	0.2851	0.054*	
C5	0.3001 (2)	−0.2975 (6)	0.3752 (3)	0.0402 (14)	
H5	0.2557	−0.3438	0.3529	0.048*	
C6	0.2170 (2)	0.0318 (6)	0.6268 (3)	0.0319 (12)	
C7	0.1856 (3)	0.0303 (7)	0.6722 (3)	0.0438 (14)	
H7	0.2122	0.0646	0.7212	0.053*	
C8	0.1214 (3)	−0.0163 (6)	0.6493 (3)	0.0486 (15)	
H8	0.1028	−0.0120	0.6805	0.058*	
C9	0.0846 (3)	−0.0697 (6)	0.5797 (3)	0.0407 (14)	
H9	0.0395	−0.1073	0.5609	0.049*	
C10	0.1149 (3)	−0.0686 (6)	0.5349 (3)	0.0409 (14)	
H10	0.0887	−0.1059	0.4861	0.049*	
C11	0.1076 (2)	0.4132 (6)	0.5198 (2)	0.0330 (13)	
C12	0.0749 (2)	0.4095 (6)	0.5644 (3)	0.0365 (13)	
H12	0.0301	0.3665	0.5432	0.044*	
C13	0.1047 (3)	0.4630 (7)	0.6326 (3)	0.0492 (15)	
H13	0.0824	0.4585	0.6602	0.059*	
C14	0.1692 (3)	0.5252 (7)	0.6615 (3)	0.0461 (15)	
H14	0.1929	0.5617	0.7105	0.055*	
C15	0.2008 (2)	0.5354 (6)	0.6187 (3)	0.0383 (13)	
H15	0.2450	0.5824	0.6396	0.046*	
C16	0.2826 (2)	0.2062 (6)	0.3712 (3)	0.0328 (12)	
C17	0.3156 (3)	0.2097 (6)	0.3269 (2)	0.0377 (13)	
H17	0.2897	0.1730	0.2781	0.045*	
C18	0.3795 (3)	0.2606 (6)	0.3499 (3)	0.0430 (14)	
H18	0.3982	0.2626	0.3189	0.052*	
C19	0.4145 (2)	0.3083 (6)	0.4196 (3)	0.0422 (14)	
H19	0.4598	0.3455	0.4390	0.051*	
C20	0.3856 (2)	0.3042 (6)	0.4644 (2)	0.0361 (14)	
H20	0.4122	0.3381	0.5134	0.043*	
C21	0.4071 (3)	0.5216 (9)	0.6919 (3)	0.104 (2)	
H21A	0.3826	0.4409	0.7088	0.125*	
H21B	0.4172	0.6405	0.7186	0.125*	
C22	0.4667 (4)	0.4349 (10)	0.7052 (4)	0.189 (4)	
H22A	0.4565	0.3245	0.6747	0.227*	
H22B	0.4918	0.3981	0.7559	0.227*	
H22C	0.4935	0.5216	0.6944	0.227*	
C23	0.0849 (4)	−0.2585 (12)	0.3053 (4)	0.145 (3)	
H23A	0.1082	−0.1707	0.2893	0.174*	
H23B	0.0715	−0.3695	0.2738	0.174*	
C24	0.0319 (4)	−0.1783 (11)	0.3032 (6)	0.220 (6)	
H24A	0.0463	−0.0711	0.3358	0.264*	
H24B	0.0090	−0.2679	0.3184	0.264*	
H24C	0.0013	−0.1367	0.2541	0.264*	
Li1	0.3025 (4)	0.0014 (11)	0.5407 (4)	0.041 (2)	
Li2	0.1956 (4)	0.2363 (11)	0.4551 (4)	0.038 (2)	
N1	0.3283 (2)	−0.2435 (5)	0.4436 (2)	0.0319 (10)	
N2	0.1786 (2)	−0.0174 (5)	0.5582 (2)	0.0278 (10)	
N3	0.1707 (2)	0.4819 (5)	0.5507 (2)	0.0324 (10)	
N4	0.3213 (2)	0.2539 (5)	0.4404 (2)	0.0297 (10)	
O1	0.29231 (15)	−0.2565 (4)	0.48172 (16)	0.0402 (9)	
O2	0.20416 (14)	−0.0166 (4)	0.51000 (15)	0.0331 (9)	
O3	0.20569 (14)	0.4927 (4)	0.51171 (15)	0.0340 (9)	
O4	0.29464 (15)	0.2503 (4)	0.48815 (15)	0.0363 (9)	
O5	0.36826 (15)	0.5544 (5)	0.61947 (16)	0.0843 (12)	
H5A	0.3835	0.6455	0.6078	0.101*	
O6	0.12663 (16)	−0.3096 (5)	0.37800 (19)	0.1027 (15)	
H6A	0.1102	−0.4016	0.3878	0.123*	
Li3	0.1999 (4)	−0.2468 (13)	0.4565 (5)	0.060 (3)	
Li4	0.2976 (5)	0.4811 (15)	0.5386 (5)	0.082 (4)	
S1	0.42243 (5)	−0.10135 (16)	0.56162 (6)	0.0439 (3)	
S2	0.29818 (6)	0.08162 (19)	0.65231 (6)	0.0561 (4)	
S3	0.07545 (5)	0.33344 (16)	0.43499 (6)	0.0461 (4)	
S4	0.20225 (6)	0.15195 (19)	0.34460 (6)	0.0591 (4)	

### Atomic displacement parameters (Å^2^)

**Table:**

	*U*^11^	*U*^22^	*U*^33^	*U*^12^	*U*^13^	*U*^23^
C1	0.023 (3)	0.025 (3)	0.041 (3)	0.004 (3)	0.017 (2)	0.003 (2)
C2	0.037 (3)	0.035 (4)	0.045 (3)	0.006 (3)	0.021 (3)	0.005 (3)
C3	0.053 (4)	0.044 (3)	0.053 (4)	0.006 (3)	0.035 (3)	0.008 (3)
C4	0.061 (4)	0.044 (3)	0.038 (3)	0.011 (3)	0.032 (3)	0.004 (3)
C5	0.036 (3)	0.031 (3)	0.034 (3)	−0.005 (3)	0.003 (3)	−0.006 (3)
C6	0.043 (3)	0.028 (3)	0.031 (3)	−0.001 (2)	0.023 (2)	0.003 (2)
C7	0.049 (3)	0.043 (3)	0.041 (3)	0.006 (3)	0.022 (3)	0.003 (3)
C8	0.059 (4)	0.043 (4)	0.068 (4)	0.003 (3)	0.049 (3)	0.006 (3)
C9	0.037 (3)	0.027 (3)	0.061 (4)	−0.001 (3)	0.026 (3)	0.001 (3)
C10	0.033 (4)	0.042 (3)	0.048 (3)	−0.008 (3)	0.020 (3)	0.001 (3)
C11	0.021 (3)	0.035 (3)	0.044 (3)	−0.002 (3)	0.017 (2)	0.001 (3)
C12	0.033 (3)	0.038 (3)	0.046 (3)	0.004 (3)	0.025 (3)	0.009 (3)
C13	0.069 (4)	0.036 (3)	0.065 (4)	0.009 (3)	0.050 (3)	0.014 (3)
C14	0.059 (4)	0.040 (3)	0.035 (3)	0.003 (3)	0.019 (3)	−0.003 (3)
C15	0.033 (3)	0.043 (3)	0.033 (3)	0.008 (3)	0.012 (3)	0.008 (3)
C16	0.031 (3)	0.038 (3)	0.027 (3)	0.004 (2)	0.012 (2)	−0.001 (3)
C17	0.041 (3)	0.039 (3)	0.031 (3)	0.004 (3)	0.017 (2)	−0.002 (3)
C18	0.050 (4)	0.050 (4)	0.040 (3)	0.009 (3)	0.031 (3)	0.009 (3)
C19	0.035 (3)	0.048 (4)	0.051 (4)	0.005 (3)	0.027 (3)	0.013 (3)
C20	0.023 (3)	0.036 (3)	0.037 (3)	−0.001 (3)	0.005 (2)	0.003 (2)
C21	0.104 (5)	0.116 (6)	0.048 (4)	−0.041 (5)	0.004 (4)	0.025 (4)
C22	0.080 (5)	0.165 (8)	0.234 (9)	0.066 (6)	0.008 (6)	0.051 (7)
C23	0.143 (7)	0.152 (8)	0.130 (7)	−0.060 (7)	0.059 (7)	0.011 (6)
C24	0.118 (7)	0.145 (8)	0.334 (13)	0.062 (7)	0.062 (8)	0.052 (8)
Li1	0.028 (5)	0.049 (6)	0.040 (5)	0.002 (4)	0.012 (4)	0.003 (4)
Li2	0.033 (5)	0.035 (5)	0.051 (6)	0.004 (4)	0.024 (4)	0.001 (4)
N1	0.027 (3)	0.028 (3)	0.040 (3)	0.001 (2)	0.016 (2)	0.003 (2)
N2	0.025 (3)	0.029 (3)	0.028 (2)	−0.001 (2)	0.011 (2)	−0.002 (2)
N3	0.028 (3)	0.038 (3)	0.031 (3)	0.005 (2)	0.014 (2)	−0.001 (2)
N4	0.031 (3)	0.023 (3)	0.035 (3)	0.002 (2)	0.016 (2)	0.002 (2)
O1	0.024 (2)	0.059 (3)	0.040 (2)	−0.0005 (17)	0.0177 (16)	−0.0005 (18)
O2	0.029 (2)	0.046 (2)	0.0271 (18)	−0.0026 (17)	0.0156 (15)	−0.0028 (16)
O3	0.023 (2)	0.046 (2)	0.0343 (19)	−0.0024 (17)	0.0147 (16)	−0.0004 (17)
O4	0.031 (2)	0.055 (2)	0.0269 (19)	0.0015 (18)	0.0175 (16)	−0.0025 (17)
O5	0.078 (2)	0.087 (3)	0.043 (2)	−0.029 (2)	−0.0056 (18)	0.015 (2)
O6	0.092 (3)	0.091 (3)	0.061 (2)	−0.029 (2)	−0.013 (2)	0.015 (2)
Li3	0.039 (6)	0.078 (7)	0.061 (6)	−0.006 (5)	0.023 (5)	−0.029 (6)
Li4	0.039 (6)	0.123 (10)	0.070 (7)	0.001 (6)	0.017 (5)	−0.031 (7)
S1	0.0304 (7)	0.0557 (9)	0.0404 (7)	−0.0041 (7)	0.0132 (5)	−0.0001 (7)
S2	0.0376 (7)	0.0886 (11)	0.0381 (7)	−0.0099 (8)	0.0155 (6)	−0.0115 (8)
S3	0.0316 (7)	0.0543 (9)	0.0453 (8)	−0.0038 (7)	0.0133 (6)	−0.0025 (7)
S4	0.0385 (7)	0.0946 (11)	0.0391 (7)	−0.0120 (8)	0.0150 (6)	−0.0158 (8)

### Geometric parameters (Å, °)

**Table:**

C1—N1	1.335 (5)	C21—H21A	0.9700
C1—C2	1.427 (6)	C21—H21B	0.9700
C1—S1	1.651 (5)	C22—H22A	0.9600
C2—C3	1.318 (6)	C22—H22B	0.9600
C2—H2	0.9300	C22—H22C	0.9600
C3—C4	1.342 (6)	C23—C24	1.301 (8)
C3—H3	0.9300	C23—O6	1.386 (7)
C4—C5	1.399 (6)	C23—H23A	0.9700
C4—H4	0.9300	C23—H23B	0.9700
C5—N1	1.304 (5)	C24—H24A	0.9600
C5—H5	0.9300	C24—H24B	0.9600
C6—N2	1.306 (5)	C24—H24C	0.9600
C6—C7	1.433 (6)	Li1—O2	1.982 (8)
C6—S2	1.670 (5)	Li1—O4	2.030 (8)
C7—C8	1.321 (6)	Li1—O1	2.144 (8)
C7—H7	0.9300	Li1—S2	2.446 (8)
C8—C9	1.328 (6)	Li1—S1	2.616 (8)
C8—H8	0.9300	Li1—Li3	2.740 (9)
C9—C10	1.401 (6)	Li1—Li2	2.740 (8)
C9—H9	0.9300	Li1—Li4	3.382 (12)
C10—N2	1.318 (5)	Li2—O4	1.986 (8)
C10—H10	0.9300	Li2—O2	2.077 (8)
C11—N3	1.331 (5)	Li2—O3	2.110 (8)
C11—C12	1.441 (6)	Li2—S4	2.457 (8)
C11—S3	1.649 (5)	Li2—S3	2.615 (8)
C12—C13	1.299 (5)	Li2—Li4	2.713 (11)
C12—H12	0.9300	Li2—Li3	3.406 (10)
C13—C14	1.343 (6)	N1—O1	1.389 (5)
C13—H13	0.9300	N2—O2	1.382 (4)
C14—C15	1.389 (6)	N3—O3	1.384 (4)
C14—H14	0.9300	N4—O4	1.392 (4)
C15—N3	1.293 (5)	O1—Li3	1.880 (9)
C15—H15	0.9300	O1—Li4^i^	2.170 (11)
C16—N4	1.314 (5)	O2—Li3	1.945 (9)
C16—C17	1.439 (6)	O3—Li4	1.860 (10)
C16—S4	1.656 (5)	O3—Li3^ii^	2.137 (10)
C17—C18	1.323 (5)	O4—Li4	1.921 (10)
C17—H17	0.9300	O5—Li4	1.737 (9)
C18—C19	1.315 (5)	O5—H5A	0.8200
C18—H18	0.9300	O6—Li3	1.718 (8)
C19—C20	1.377 (6)	O6—H6A	0.8200
C19—H19	0.9300	Li3—O3^i^	2.137 (10)
C20—N4	1.326 (5)	Li3—Li4^i^	2.785 (11)
C20—H20	0.9300	Li4—O1^ii^	2.170 (11)
C21—O5	1.346 (5)	Li4—Li3^ii^	2.785 (11)
C21—C22	1.372 (7)		
			
Cg1···Cg2	3.470 (6)	Cg3···Cg4	3.461 (6)
Cg1···Cg2^ii^	3.596 (6)	Cg3···Cg4^ii^	3.607 (6)
Cg2···Cg1^i^	3.596 (6)	Cg4···Cg3^i^	3.607 (6)
			
N1—C1—C2	116.9 (4)	O2—Li1—S1	158.8 (4)
N1—C1—S1	116.2 (4)	O4—Li1—S1	98.0 (3)
C2—C1—S1	126.9 (4)	O1—Li1—S1	70.9 (2)
N1—C1—C20	108.7 (3)	S2—Li1—S1	115.1 (3)
C2—C1—C20	86.5 (3)	O2—Li1—Li3	45.2 (2)
S1—C1—C20	74.97 (17)	O4—Li1—Li3	112.4 (3)
C3—C2—C1	124.7 (5)	O1—Li1—Li3	43.2 (2)
C3—C2—C19	102.6 (3)	S2—Li1—Li3	110.2 (3)
C1—C2—C19	92.6 (3)	S1—Li1—Li3	114.0 (3)
C3—C2—H2	117.6	O2—Li1—Li2	49.0 (2)
C1—C2—H2	117.6	O4—Li1—Li2	46.3 (2)
C19—C2—H2	73.4	O1—Li1—Li2	109.0 (3)
C2—C3—C4	115.9 (5)	S2—Li1—Li2	92.9 (3)
C2—C3—C18	77.3 (3)	S1—Li1—Li2	141.3 (3)
C4—C3—C18	104.0 (3)	Li3—Li1—Li2	76.9 (2)
C2—C3—H3	122.0	O2—Li1—Li4	92.1 (3)
C4—C3—H3	122.0	O4—Li1—Li4	30.2 (2)
C18—C3—H3	88.8	O1—Li1—Li4	147.8 (3)
C3—C4—C5	120.3 (5)	S2—Li1—Li4	76.4 (2)
C3—C4—C17	75.9 (3)	S1—Li1—Li4	107.7 (2)
C5—C4—C17	89.0 (3)	Li3—Li1—Li4	128.2 (3)
C3—C4—H4	119.9	Li2—Li1—Li4	51.3 (2)
C5—C4—H4	119.9	O4—Li2—O2	93.8 (3)
C17—C4—H4	105.2	O4—Li2—O3	88.2 (3)
N1—C5—C4	122.9 (5)	O2—Li2—O3	118.1 (4)
N1—C5—C16	74.0 (3)	O4—Li2—S4	76.4 (3)
C4—C5—C16	91.6 (3)	O2—Li2—S4	106.2 (3)
N1—C5—H5	118.6	O3—Li2—S4	134.0 (4)
C4—C5—H5	118.6	O4—Li2—S3	159.8 (4)
C16—C5—H5	105.0	O2—Li2—S3	97.0 (3)
N2—C6—C7	116.6 (5)	O3—Li2—S3	71.6 (2)
N2—C6—S2	116.0 (4)	S4—Li2—S3	116.5 (3)
C7—C6—S2	127.4 (4)	O4—Li2—Li4	45.0 (3)
N2—C6—C15	102.4 (3)	O2—Li2—Li4	111.7 (3)
C7—C6—C15	88.1 (3)	O3—Li2—Li4	43.1 (3)
S2—C6—C15	82.90 (18)	S4—Li2—Li4	109.7 (3)
C8—C7—C6	124.6 (5)	S3—Li2—Li4	114.8 (3)
C8—C7—C14	99.4 (3)	O4—Li2—Li1	47.7 (2)
C6—C7—C14	91.2 (3)	O2—Li2—Li1	46.1 (2)
C8—C7—H7	117.7	O3—Li2—Li1	109.4 (3)
C6—C7—H7	117.7	S4—Li2—Li1	91.7 (2)
C14—C7—H7	78.6	S3—Li2—Li1	140.4 (3)
C7—C8—C9	116.8 (5)	Li4—Li2—Li1	76.7 (3)
C7—C8—C13	80.6 (3)	O4—Li2—Li3	91.4 (3)
C9—C8—C13	101.0 (3)	O2—Li2—Li3	31.0 (2)
C7—C8—H8	121.6	O3—Li2—Li3	148.9 (3)
C9—C8—H8	121.6	S4—Li2—Li3	75.6 (2)
C13—C8—H8	88.5	S3—Li2—Li3	106.6 (2)
C8—C9—C10	118.9 (5)	Li4—Li2—Li3	128.2 (3)
C8—C9—C12	78.3 (3)	Li1—Li2—Li3	51.57 (19)
C10—C9—C12	87.8 (3)	C5—N1—C1	119.3 (4)
C8—C9—H9	120.5	C5—N1—O1	120.0 (4)
C10—C9—H9	120.5	C1—N1—O1	120.6 (4)
C12—C9—H9	103.7	C5—N1—N4	106.1 (3)
N2—C10—C9	123.4 (5)	C1—N1—N4	71.5 (2)
N2—C10—C11	75.9 (3)	O1—N1—N4	92.5 (2)
C9—C10—C11	92.9 (3)	C6—N2—C10	119.5 (4)
N2—C10—H10	118.3	C6—N2—O2	120.7 (4)
C9—C10—H10	118.3	C10—N2—O2	119.7 (4)
C11—C10—H10	101.8	C6—N2—N3	77.0 (3)
N3—C11—C12	117.0 (4)	C10—N2—N3	103.6 (3)
N3—C11—S3	116.6 (4)	O2—N2—N3	89.6 (2)
C12—C11—S3	126.4 (4)	C15—N3—C11	119.9 (4)
N3—C11—C10	109.0 (3)	C15—N3—O3	119.2 (4)
C12—C11—C10	86.5 (3)	C11—N3—O3	120.9 (4)
S3—C11—C10	74.68 (17)	C15—N3—N2	104.8 (3)
C13—C12—C11	123.5 (5)	C11—N3—N2	71.1 (3)
C13—C12—C9	102.2 (3)	O3—N3—N2	92.9 (2)
C11—C12—C9	92.8 (3)	C16—N4—C20	119.6 (4)
C13—C12—H12	118.3	C16—N4—O4	119.9 (4)
C11—C12—H12	118.3	C20—N4—O4	120.5 (4)
C9—C12—H12	74.0	C16—N4—N1	76.4 (3)
C12—C13—C14	116.8 (5)	C20—N4—N1	103.2 (3)
C12—C13—C8	78.2 (3)	O4—N4—N1	90.0 (2)
C14—C13—C8	103.7 (3)	N1—O1—Li3	135.4 (4)
C12—C13—H13	121.6	N1—O1—Li1	110.2 (3)
C14—C13—H13	121.6	Li3—O1—Li1	85.6 (3)
C8—C13—H13	88.2	N1—O1—Li4^i^	118.6 (4)
C13—C14—C15	120.6 (5)	Li3—O1—Li4^i^	86.6 (4)
C13—C14—C7	76.2 (3)	Li1—O1—Li4^i^	116.5 (4)
C15—C14—C7	91.3 (3)	N2—O2—Li3	119.6 (4)
C13—C14—H14	119.7	N2—O2—Li1	124.1 (3)
C15—C14—H14	119.7	Li3—O2—Li1	88.5 (4)
C7—C14—H14	102.7	N2—O2—Li2	116.5 (3)
N3—C15—C14	122.3 (5)	Li3—O2—Li2	115.7 (4)
N3—C15—C6	75.4 (3)	Li1—O2—Li2	84.9 (3)
C14—C15—C6	89.4 (3)	N3—O3—Li4	133.4 (4)
N3—C15—H15	118.9	N3—O3—Li2	110.3 (3)
C14—C15—H15	118.9	Li4—O3—Li2	86.0 (4)
C6—C15—H15	105.7	N3—O3—Li3^ii^	117.6 (3)
N4—C16—C17	115.5 (4)	Li4—O3—Li3^ii^	88.1 (4)
N4—C16—S4	116.9 (4)	Li2—O3—Li3^ii^	118.1 (3)
C17—C16—S4	127.5 (4)	N4—O4—Li4	117.8 (4)
N4—C16—C5	102.9 (3)	N4—O4—Li2	123.7 (3)
C17—C16—C5	87.0 (3)	Li4—O4—Li2	88.0 (4)
S4—C16—C5	82.39 (18)	N4—O4—Li1	116.6 (3)
C18—C17—C16	125.5 (5)	Li4—O4—Li1	117.7 (4)
C18—C17—C4	99.9 (3)	Li2—O4—Li1	86.0 (3)
C16—C17—C4	92.4 (3)	C21—O5—Li4	145.4 (5)
C18—C17—H17	117.2	C21—O5—H5A	109.5
C16—C17—H17	117.2	Li4—O5—H5A	105.1
C4—C17—H17	76.4	C23—O6—Li3	142.8 (5)
C19—C18—C17	115.3 (5)	C23—O6—H6A	109.5
C19—C18—C3	100.1 (3)	Li3—O6—H6A	107.7
C17—C18—C3	79.9 (3)	O6—Li3—O1	132.1 (5)
C19—C18—H18	122.4	O6—Li3—O2	121.2 (5)
C17—C18—H18	122.4	O1—Li3—O2	97.6 (4)
C3—C18—H18	90.0	O6—Li3—O3^i^	94.0 (4)
C18—C19—C20	121.3 (5)	O1—Li3—O3^i^	92.9 (4)
C18—C19—C2	79.6 (3)	O2—Li3—O3^i^	115.7 (4)
C20—C19—C2	87.7 (3)	O6—Li3—Li1	152.8 (5)
C18—C19—H19	119.4	O1—Li3—Li1	51.3 (3)
C20—C19—H19	119.4	O2—Li3—Li1	46.3 (3)
C2—C19—H19	103.0	O3^i^—Li3—Li1	113.1 (3)
N4—C20—C19	122.7 (4)	O6—Li3—Li4^i^	120.6 (4)
N4—C20—C1	76.1 (3)	O1—Li3—Li4^i^	51.0 (3)
C19—C20—C1	93.1 (3)	O2—Li3—Li4^i^	114.7 (4)
N4—C20—H20	118.6	O3^i^—Li3—Li4^i^	41.9 (3)
C19—C20—H20	118.6	Li1—Li3—Li4^i^	83.2 (3)
C1—C20—H20	101.2	O6—Li3—Li2	103.9 (4)
O5—C21—C22	109.6 (6)	O1—Li3—Li2	93.5 (3)
O5—C21—H21A	109.7	O2—Li3—Li2	33.3 (2)
C22—C21—H21A	109.7	O3^i^—Li3—Li2	149.0 (3)
O5—C21—H21B	109.7	Li1—Li3—Li2	51.57 (19)
C22—C21—H21B	109.7	Li4^i^—Li3—Li2	134.7 (3)
H21A—C21—H21B	108.2	O5—Li4—O3	129.3 (6)
C21—C22—H22A	109.5	O5—Li4—O4	123.7 (5)
C21—C22—H22B	109.5	O3—Li4—O4	97.9 (4)
H22A—C22—H22B	109.5	O5—Li4—O1^ii^	93.3 (5)
C21—C22—H22C	109.5	O3—Li4—O1^ii^	92.4 (4)
H22A—C22—H22C	109.5	O4—Li4—O1^ii^	116.3 (5)
H22B—C22—H22C	109.5	O5—Li4—Li2	153.8 (6)
C24—C23—O6	105.5 (8)	O3—Li4—Li2	50.9 (3)
C24—C23—H23A	110.7	O4—Li4—Li2	47.0 (3)
O6—C23—H23A	110.7	O1^ii^—Li4—Li2	112.8 (4)
C24—C23—H23B	110.6	O5—Li4—Li3^ii^	117.7 (5)
O6—C23—H23B	110.6	O3—Li4—Li3^ii^	50.1 (3)
H23A—C23—H23B	108.8	O4—Li4—Li3^ii^	116.1 (4)
C23—C24—H24A	109.4	O1^ii^—Li4—Li3^ii^	42.4 (3)
C23—C24—H24B	109.5	Li2—Li4—Li3^ii^	83.0 (3)
H24A—C24—H24B	109.5	O5—Li4—Li1	106.0 (4)
C23—C24—H24C	109.5	O3—Li4—Li1	94.1 (4)
H24A—C24—H24C	109.5	O4—Li4—Li1	32.1 (3)
H24B—C24—H24C	109.5	O1^ii^—Li4—Li1	148.4 (4)
O2—Li1—O4	95.3 (3)	Li2—Li4—Li1	52.0 (2)
O2—Li1—O1	88.4 (3)	Li3^ii^—Li4—Li1	135.0 (3)
O4—Li1—O1	117.7 (4)	C1—S1—Li1	93.3 (2)
O2—Li1—S2	76.4 (3)	C6—S2—Li1	101.2 (2)
O4—Li1—S2	106.4 (3)	C11—S3—Li2	92.4 (2)
O1—Li1—S2	134.5 (4)	C16—S4—Li2	100.8 (2)
			
N1—C1—C2—C3	0.6 (7)	O2—Li2—O3—Li4	92.6 (5)
S1—C1—C2—C3	−176.9 (4)	S4—Li2—O3—Li4	−69.8 (6)
C20—C1—C2—C3	−108.6 (5)	S3—Li2—O3—Li4	−179.2 (3)
N1—C1—C2—C19	108.0 (4)	Li1—Li2—O3—Li4	42.7 (4)
S1—C1—C2—C19	−69.5 (4)	Li3—Li2—O3—Li4	89.0 (6)
C20—C1—C2—C19	−1.25 (14)	O4—Li2—O3—Li3^ii^	84.9 (4)
C1—C2—C3—C4	−0.1 (8)	O2—Li2—O3—Li3^ii^	178.2 (3)
C19—C2—C3—C4	−102.3 (4)	S4—Li2—O3—Li3^ii^	15.9 (7)
C1—C2—C3—C18	99.5 (5)	S3—Li2—O3—Li3^ii^	−93.5 (4)
C19—C2—C3—C18	−2.76 (16)	Li4—Li2—O3—Li3^ii^	85.7 (5)
C2—C3—C4—C5	−0.4 (7)	Li1—Li2—O3—Li3^ii^	128.4 (4)
C18—C3—C4—C5	−83.0 (5)	Li3—Li2—O3—Li3^ii^	174.6 (5)
C2—C3—C4—C17	79.9 (4)	C16—N4—O4—Li4	−122.7 (5)
C18—C3—C4—C17	−2.60 (16)	C20—N4—O4—Li4	57.7 (6)
C3—C4—C5—N1	0.4 (8)	N1—N4—O4—Li4	163.1 (3)
C17—C4—C5—N1	−72.6 (5)	C16—N4—O4—Li2	−15.0 (6)
C3—C4—C5—C16	72.4 (5)	C20—N4—O4—Li2	165.3 (4)
C17—C4—C5—C16	−0.62 (14)	N1—N4—O4—Li2	−89.3 (4)
N2—C6—C7—C8	−0.2 (8)	C16—N4—O4—Li1	88.9 (5)
S2—C6—C7—C8	−177.7 (4)	C20—N4—O4—Li1	−90.7 (5)
C15—C6—C7—C8	102.7 (5)	N1—N4—O4—Li1	14.7 (3)
N2—C6—C7—C14	−102.4 (4)	O2—Li2—O4—N4	120.2 (4)
S2—C6—C7—C14	80.1 (4)	O3—Li2—O4—N4	−121.8 (3)
C15—C6—C7—C14	0.49 (14)	S4—Li2—O4—N4	14.5 (4)
C6—C7—C8—C9	1.7 (8)	S3—Li2—O4—N4	−117.5 (10)
C14—C7—C8—C9	99.8 (4)	Li4—Li2—O4—N4	−122.5 (5)
C6—C7—C8—C13	−95.9 (5)	Li1—Li2—O4—N4	119.6 (4)
C14—C7—C8—C13	2.11 (16)	Li3—Li2—O4—N4	89.3 (4)
C7—C8—C9—C10	−1.7 (7)	O2—Li2—O4—Li4	−117.3 (4)
C13—C8—C9—C10	83.2 (5)	O3—Li2—O4—Li4	0.7 (4)
C7—C8—C9—C12	−82.5 (4)	S4—Li2—O4—Li4	137.0 (3)
C13—C8—C9—C12	2.41 (15)	S3—Li2—O4—Li4	5.0 (12)
C8—C9—C10—N2	0.2 (8)	Li1—Li2—O4—Li4	−117.9 (4)
C12—C9—C10—N2	75.5 (5)	Li3—Li2—O4—Li4	−148.2 (4)
C8—C9—C10—C11	−74.8 (4)	O2—Li2—O4—Li1	0.6 (4)
C12—C9—C10—C11	0.57 (14)	O3—Li2—O4—Li1	118.6 (3)
N2—C10—C11—N3	−7.8 (3)	S4—Li2—O4—Li1	−105.1 (2)
C9—C10—C11—N3	116.0 (4)	S3—Li2—O4—Li1	122.9 (11)
N2—C10—C11—C12	−125.1 (4)	Li4—Li2—O4—Li1	117.9 (4)
C9—C10—C11—C12	−1.3 (3)	Li3—Li2—O4—Li1	−30.3 (3)
N2—C10—C11—S3	105.6 (3)	O2—Li1—O4—N4	−126.6 (3)
C9—C10—C11—S3	−130.6 (4)	O1—Li1—O4—N4	−35.7 (5)
N3—C11—C12—C13	−2.4 (7)	S2—Li1—O4—N4	156.0 (3)
S3—C11—C12—C13	175.2 (4)	S1—Li1—O4—N4	36.9 (4)
C10—C11—C12—C13	107.2 (5)	Li3—Li1—O4—N4	−83.3 (4)
N3—C11—C12—C9	−109.0 (4)	Li2—Li1—O4—N4	−126.0 (4)
S3—C11—C12—C9	68.6 (4)	Li4—Li1—O4—N4	148.4 (5)
C10—C11—C12—C9	0.55 (14)	O2—Li1—O4—Li4	84.9 (4)
C8—C9—C12—C13	−6.4 (4)	O1—Li1—O4—Li4	175.9 (3)
C10—C9—C12—C13	−126.5 (5)	S2—Li1—O4—Li4	7.6 (6)
C8—C9—C12—C11	118.8 (5)	S1—Li1—O4—Li4	−111.6 (4)
C10—C9—C12—C11	−1.3 (3)	Li3—Li1—O4—Li4	128.3 (4)
C11—C12—C13—C14	0.3 (8)	Li2—Li1—O4—Li4	85.6 (5)
C9—C12—C13—C14	102.0 (4)	O2—Li1—O4—Li2	−0.7 (4)
C11—C12—C13—C8	−99.2 (5)	O1—Li1—O4—Li2	90.3 (4)
C9—C12—C13—C8	2.48 (16)	S2—Li1—O4—Li2	−78.0 (3)
C7—C8—C13—C12	109.4 (5)	S1—Li1—O4—Li2	162.8 (2)
C9—C8—C13—C12	−6.3 (4)	Li3—Li1—O4—Li2	42.7 (4)
C7—C8—C13—C14	−5.6 (4)	Li4—Li1—O4—Li2	−85.6 (5)
C9—C8—C13—C14	−121.3 (5)	C22—C21—O5—Li4	103.7 (9)
C12—C13—C14—C15	1.8 (8)	C24—C23—O6—Li3	−104.1 (9)
C8—C13—C14—C15	85.3 (5)	C23—O6—Li3—O1	−69.8 (12)
C12—C13—C14—C7	−81.4 (4)	C23—O6—Li3—O2	69.2 (11)
C8—C13—C14—C7	2.15 (16)	C23—O6—Li3—O3^i^	−167.4 (8)
C8—C7—C14—C13	−5.4 (4)	C23—O6—Li3—Li1	15.3 (16)
C6—C7—C14—C13	120.0 (5)	C23—O6—Li3—Li4^i^	−133.0 (9)
C8—C7—C14—C15	−126.7 (5)	C23—O6—Li3—Li2	38.2 (10)
C6—C7—C14—C15	−1.2 (4)	N1—O1—Li3—O6	29.8 (11)
C13—C14—C15—N3	−1.8 (8)	Li1—O1—Li3—O6	144.3 (7)
C7—C14—C15—N3	72.8 (5)	Li4^i^—O1—Li3—O6	−98.7 (7)
C13—C14—C15—C6	−74.1 (5)	N1—O1—Li3—O2	−115.7 (5)
C7—C14—C15—C6	0.50 (14)	Li1—O1—Li3—O2	−1.2 (4)
N2—C6—C15—N3	−8.0 (3)	Li4^i^—O1—Li3—O2	115.8 (4)
C7—C6—C15—N3	−124.9 (4)	N1—O1—Li3—O3^i^	127.9 (5)
S2—C6—C15—N3	107.1 (4)	Li1—O1—Li3—O3^i^	−117.6 (3)
N2—C6—C15—C14	115.6 (4)	Li4^i^—O1—Li3—O3^i^	−0.6 (4)
C7—C6—C15—C14	−1.2 (4)	N1—O1—Li3—Li1	−114.5 (6)
S2—C6—C15—C14	−129.3 (4)	Li4^i^—O1—Li3—Li1	116.9 (3)
N1—C5—C16—N4	9.9 (4)	N1—O1—Li3—Li4^i^	128.6 (6)
C4—C5—C16—N4	−114.0 (4)	Li1—O1—Li3—Li4^i^	−116.9 (3)
N1—C5—C16—C17	125.4 (4)	N1—O1—Li3—Li2	−82.4 (5)
C4—C5—C16—C17	1.5 (3)	Li1—O1—Li3—Li2	32.1 (3)
N1—C5—C16—S4	−106.1 (4)	Li4^i^—O1—Li3—Li2	149.0 (3)
C4—C5—C16—S4	130.0 (4)	N2—O2—Li3—O6	81.6 (7)
N4—C16—C17—C18	−1.8 (8)	Li1—O2—Li3—O6	−149.3 (5)
S4—C16—C17—C18	177.4 (4)	Li2—O2—Li3—O6	−65.8 (7)
C5—C16—C17—C18	−104.6 (5)	N2—O2—Li3—O1	−127.8 (4)
N4—C16—C17—C4	102.2 (4)	Li1—O2—Li3—O1	1.3 (5)
S4—C16—C17—C4	−78.6 (4)	Li2—O2—Li3—O1	84.8 (5)
C5—C16—C17—C4	−0.60 (14)	N2—O2—Li3—O3^i^	−30.8 (6)
C3—C4—C17—C18	6.7 (4)	Li1—O2—Li3—O3^i^	98.2 (4)
C5—C4—C17—C18	128.3 (4)	Li2—O2—Li3—O3^i^	−178.2 (3)
C3—C4—C17—C16	−120.1 (5)	N2—O2—Li3—Li1	−129.0 (4)
C5—C4—C17—C16	1.5 (3)	Li2—O2—Li3—Li1	83.6 (4)
C16—C17—C18—C19	1.1 (8)	N2—O2—Li3—Li4^i^	−77.3 (4)
C4—C17—C18—C19	−99.1 (4)	Li1—O2—Li3—Li4^i^	51.7 (4)
C16—C17—C18—C3	97.7 (5)	Li2—O2—Li3—Li4^i^	135.3 (4)
C4—C17—C18—C3	−2.56 (16)	N2—O2—Li3—Li2	147.4 (5)
C2—C3—C18—C19	7.1 (4)	Li1—O2—Li3—Li2	−83.6 (4)
C4—C3—C18—C19	121.0 (5)	O2—Li1—Li3—O6	72.9 (9)
C2—C3—C18—C17	−107.1 (5)	O4—Li1—Li3—O6	−1.5 (10)
C4—C3—C18—C17	6.8 (4)	O1—Li1—Li3—O6	−108.7 (10)
C17—C18—C19—C20	0.0 (7)	S2—Li1—Li3—O6	117.0 (9)
C3—C18—C19—C20	−83.5 (5)	S1—Li1—Li3—O6	−111.9 (9)
C17—C18—C19—C2	80.8 (4)	Li2—Li1—Li3—O6	28.8 (9)
C3—C18—C19—C2	−2.72 (15)	Li4—Li1—Li3—O6	28.7 (11)
C3—C2—C19—C18	7.2 (4)	O2—Li1—Li3—O1	−178.4 (6)
C1—C2—C19—C18	−119.3 (5)	O4—Li1—Li3—O1	107.2 (4)
C3—C2—C19—C20	129.6 (5)	S2—Li1—Li3—O1	−134.3 (4)
C1—C2—C19—C20	3.1 (3)	S1—Li1—Li3—O1	−3.2 (3)
C18—C19—C20—N4	−0.4 (8)	Li2—Li1—Li3—O1	137.4 (4)
C2—C19—C20—N4	−76.8 (4)	Li4—Li1—Li3—O1	137.3 (4)
C18—C19—C20—C1	75.0 (5)	O4—Li1—Li3—O2	−74.4 (4)
C2—C19—C20—C1	−1.30 (14)	O1—Li1—Li3—O2	178.4 (6)
N1—C1—C20—N4	8.8 (3)	S2—Li1—Li3—O2	44.1 (3)
C2—C1—C20—N4	126.1 (4)	S1—Li1—Li3—O2	175.2 (5)
S1—C1—C20—N4	−104.2 (3)	Li2—Li1—Li3—O2	−44.2 (3)
N1—C1—C20—C19	−114.2 (4)	Li4—Li1—Li3—O2	−44.3 (4)
C2—C1—C20—C19	3.1 (3)	O2—Li1—Li3—O3^i^	−104.1 (5)
S1—C1—C20—C19	132.8 (4)	O4—Li1—Li3—O3^i^	−178.6 (4)
O2—Li1—Li2—O4	179.1 (5)	O1—Li1—Li3—O3^i^	74.2 (4)
O1—Li1—Li2—O4	−110.6 (4)	S2—Li1—Li3—O3^i^	−60.1 (4)
S2—Li1—Li2—O4	110.0 (4)	S1—Li1—Li3—O3^i^	71.0 (4)
S1—Li1—Li2—O4	−27.8 (4)	Li2—Li1—Li3—O3^i^	−148.3 (4)
Li3—Li1—Li2—O4	−139.9 (3)	Li4—Li1—Li3—O3^i^	−148.4 (3)
Li4—Li1—Li2—O4	40.0 (3)	O2—Li1—Li3—Li4^i^	−134.1 (4)
O4—Li1—Li2—O2	−179.1 (5)	O4—Li1—Li3—Li4^i^	151.5 (3)
O1—Li1—Li2—O2	70.2 (3)	O1—Li1—Li3—Li4^i^	44.3 (3)
S2—Li1—Li2—O2	−69.1 (3)	S2—Li1—Li3—Li4^i^	−90.0 (3)
S1—Li1—Li2—O2	153.0 (6)	S1—Li1—Li3—Li4^i^	41.1 (3)
Li3—Li1—Li2—O2	40.9 (3)	Li2—Li1—Li3—Li4^i^	−178.3 (3)
Li4—Li1—Li2—O2	−139.1 (3)	Li4—Li1—Li3—Li4^i^	−178.4 (4)
O2—Li1—Li2—O3	110.7 (4)	O2—Li1—Li3—Li2	44.2 (3)
O4—Li1—Li2—O3	−68.5 (3)	O4—Li1—Li3—Li2	−30.2 (3)
O1—Li1—Li2—O3	−179.1 (4)	O1—Li1—Li3—Li2	−137.4 (4)
S2—Li1—Li2—O3	41.5 (3)	S2—Li1—Li3—Li2	88.3 (3)
S1—Li1—Li2—O3	−96.3 (5)	S1—Li1—Li3—Li2	−140.6 (3)
Li3—Li1—Li2—O3	151.6 (3)	Li4—Li1—Li3—Li2	−0.1 (3)
Li4—Li1—Li2—O3	−28.5 (3)	O4—Li2—Li3—O6	−138.5 (5)
O2—Li1—Li2—S4	−111.0 (3)	O2—Li2—Li3—O6	126.5 (6)
O4—Li1—Li2—S4	69.8 (3)	O3—Li2—Li3—O6	132.7 (7)
O1—Li1—Li2—S4	−40.8 (3)	S4—Li2—Li3—O6	−62.9 (3)
S2—Li1—Li2—S4	179.8 (3)	S3—Li2—Li3—O6	50.9 (4)
S1—Li1—Li2—S4	42.0 (5)	Li4—Li2—Li3—O6	−166.8 (4)
Li3—Li1—Li2—S4	−70.1 (2)	Li1—Li2—Li3—O6	−166.9 (4)
Li4—Li1—Li2—S4	109.8 (3)	O4—Li2—Li3—O1	−3.5 (3)
O2—Li1—Li2—S3	26.3 (4)	O2—Li2—Li3—O1	−98.5 (5)
O4—Li1—Li2—S3	−152.9 (6)	O3—Li2—Li3—O1	−92.3 (6)
O1—Li1—Li2—S3	96.5 (5)	S4—Li2—Li3—O1	72.1 (3)
S2—Li1—Li2—S3	−42.9 (5)	S3—Li2—Li3—O1	−174.1 (4)
S1—Li1—Li2—S3	179.3 (4)	Li4—Li2—Li3—O1	−31.8 (5)
Li3—Li1—Li2—S3	67.2 (5)	Li1—Li2—Li3—O1	−31.9 (3)
Li4—Li1—Li2—S3	−112.9 (5)	O4—Li2—Li3—O2	95.0 (4)
O2—Li1—Li2—Li4	139.1 (3)	O3—Li2—Li3—O2	6.2 (5)
O4—Li1—Li2—Li4	−40.0 (3)	S4—Li2—Li3—O2	170.5 (5)
O1—Li1—Li2—Li4	−150.6 (3)	S3—Li2—Li3—O2	−75.7 (4)
S2—Li1—Li2—Li4	70.0 (3)	Li4—Li2—Li3—O2	66.6 (5)
S1—Li1—Li2—Li4	−67.8 (5)	Li1—Li2—Li3—O2	66.5 (4)
Li3—Li1—Li2—Li4	−179.9 (3)	O4—Li2—Li3—O3^i^	98.0 (6)
O2—Li1—Li2—Li3	−40.9 (3)	O2—Li2—Li3—O3^i^	3.1 (5)
O4—Li1—Li2—Li3	139.9 (3)	O3—Li2—Li3—O3^i^	9.2 (9)
O1—Li1—Li2—Li3	29.3 (3)	S4—Li2—Li3—O3^i^	173.6 (7)
S2—Li1—Li2—Li3	−110.1 (3)	S3—Li2—Li3—O3^i^	−72.6 (7)
S1—Li1—Li2—Li3	112.1 (5)	Li4—Li2—Li3—O3^i^	69.7 (8)
Li4—Li1—Li2—Li3	179.9 (3)	Li1—Li2—Li3—O3^i^	69.6 (6)
C4—C5—N1—C1	0.1 (7)	O4—Li2—Li3—Li1	28.4 (3)
C16—C5—N1—C1	−81.2 (4)	O2—Li2—Li3—Li1	−66.5 (4)
C4—C5—N1—O1	−179.8 (4)	O3—Li2—Li3—Li1	−60.4 (6)
C16—C5—N1—O1	98.9 (4)	S4—Li2—Li3—Li1	104.0 (3)
C4—C5—N1—N4	77.6 (5)	S3—Li2—Li3—Li1	−142.2 (3)
C16—C5—N1—N4	−3.73 (13)	Li4—Li2—Li3—Li1	0.1 (4)
C2—C1—N1—C5	−0.6 (6)	O4—Li2—Li3—Li4^i^	30.8 (5)
S1—C1—N1—C5	177.1 (3)	O2—Li2—Li3—Li4^i^	−64.2 (5)
C20—C1—N1—C5	95.1 (4)	O3—Li2—Li3—Li4^i^	−58.0 (7)
C2—C1—N1—O1	179.3 (4)	S4—Li2—Li3—Li4^i^	106.4 (4)
S1—C1—N1—O1	−3.0 (6)	S3—Li2—Li3—Li4^i^	−139.8 (4)
C20—C1—N1—O1	−85.0 (4)	Li4—Li2—Li3—Li4^i^	2.5 (5)
C2—C1—N1—N4	−99.1 (4)	Li1—Li2—Li3—Li4^i^	2.4 (4)
S1—C1—N1—N4	78.6 (3)	C21—O5—Li4—O3	68.5 (13)
C20—C1—N1—N4	−3.40 (13)	C21—O5—Li4—O4	−70.7 (11)
C7—C6—N2—C10	−1.4 (7)	C21—O5—Li4—O1^ii^	164.4 (7)
S2—C6—N2—C10	176.4 (3)	C21—O5—Li4—Li2	−10.9 (17)
C15—C6—N2—C10	−95.5 (4)	C21—O5—Li4—Li3^ii^	128.1 (8)
C7—C6—N2—O2	178.8 (4)	C21—O5—Li4—Li1	−40.9 (9)
S2—C6—N2—O2	−3.4 (6)	N3—O3—Li4—O5	−31.6 (11)
C15—C6—N2—O2	84.6 (4)	Li2—O3—Li4—O5	−146.0 (8)
C7—C6—N2—N3	97.1 (4)	Li3^ii^—O3—Li4—O5	95.6 (8)
S2—C6—N2—N3	−85.1 (3)	N3—O3—Li4—O4	115.1 (5)
C15—C6—N2—N3	2.92 (12)	Li2—O3—Li4—O4	0.8 (5)
C9—C10—N2—C6	1.4 (7)	Li3^ii^—O3—Li4—O4	−117.6 (4)
C11—C10—N2—C6	85.5 (4)	N3—O3—Li4—O1^ii^	−127.9 (4)
C9—C10—N2—O2	−178.7 (4)	Li2—O3—Li4—O1^ii^	117.8 (3)
C11—C10—N2—O2	−94.7 (4)	Li3^ii^—O3—Li4—O1^ii^	−0.6 (4)
C9—C10—N2—N3	−81.2 (5)	N3—O3—Li4—Li2	114.3 (6)
C11—C10—N2—N3	2.85 (12)	Li3^ii^—O3—Li4—Li2	−118.4 (3)
C14—C15—N3—C11	−0.4 (7)	N3—O3—Li4—Li3^ii^	−127.3 (6)
C6—C15—N3—C11	79.5 (4)	Li2—O3—Li4—Li3^ii^	118.4 (3)
C14—C15—N3—O3	−178.8 (4)	N3—O3—Li4—Li1	83.0 (5)
C6—C15—N3—O3	−99.0 (4)	Li2—O3—Li4—Li1	−31.3 (3)
C14—C15—N3—N2	−76.8 (5)	Li3^ii^—O3—Li4—Li1	−149.7 (3)
C6—C15—N3—N2	3.00 (13)	N4—O4—Li4—O5	−84.0 (8)
C12—C11—N3—C15	2.3 (7)	Li2—O4—Li4—O5	148.5 (6)
S3—C11—N3—C15	−175.5 (3)	Li1—O4—Li4—O5	64.1 (8)
C10—C11—N3—C15	−93.6 (4)	N4—O4—Li4—O3	126.7 (4)
C12—C11—N3—O3	−179.3 (4)	Li2—O4—Li4—O3	−0.8 (5)
S3—C11—N3—O3	2.9 (6)	Li1—O4—Li4—O3	−85.2 (5)
C10—C11—N3—O3	84.8 (4)	N4—O4—Li4—O1^ii^	30.0 (6)
C12—C11—N3—N2	98.9 (4)	Li2—O4—Li4—O1^ii^	−97.5 (5)
S3—C11—N3—N2	−79.0 (3)	Li1—O4—Li4—O1^ii^	178.1 (3)
C10—C11—N3—N2	2.98 (13)	N4—O4—Li4—Li2	127.5 (4)
C6—N2—N3—C15	−8.2 (3)	Li1—O4—Li4—Li2	−84.4 (4)
C10—N2—N3—C15	109.5 (4)	N4—O4—Li4—Li3^ii^	77.5 (5)
O2—N2—N3—C15	−129.9 (4)	Li2—O4—Li4—Li3^ii^	−50.0 (4)
C6—N2—N3—C11	−125.2 (4)	Li1—O4—Li4—Li3^ii^	−134.4 (4)
C10—N2—N3—C11	−7.5 (3)	N4—O4—Li4—Li1	−148.1 (5)
O2—N2—N3—C11	113.1 (4)	Li2—O4—Li4—Li1	84.4 (4)
C6—N2—N3—O3	113.1 (4)	O4—Li2—Li4—O5	−79.7 (11)
C10—N2—N3—O3	−129.2 (4)	O2—Li2—Li4—O5	−7.0 (11)
O2—N2—N3—O3	−8.6 (2)	O3—Li2—Li4—O5	101.3 (12)
C17—C16—N4—C20	1.3 (6)	S4—Li2—Li4—O5	−124.5 (11)
S4—C16—N4—C20	−178.0 (3)	S3—Li2—Li4—O5	102.2 (11)
C5—C16—N4—C20	94.1 (4)	Li1—Li2—Li4—O5	−37.5 (11)
C17—C16—N4—O4	−178.4 (4)	Li3—Li2—Li4—O5	−37.6 (12)
S4—C16—N4—O4	2.3 (6)	O4—Li2—Li4—O3	179.0 (6)
C5—C16—N4—O4	−85.6 (4)	O2—Li2—Li4—O3	−108.4 (5)
C17—C16—N4—N1	−96.4 (4)	S4—Li2—Li4—O3	134.2 (4)
S4—C16—N4—N1	84.3 (3)	S3—Li2—Li4—O3	0.8 (3)
C5—C16—N4—N1	−3.61 (13)	Li1—Li2—Li4—O3	−138.9 (4)
C19—C20—N4—C16	−0.3 (7)	Li3—Li2—Li4—O3	−139.0 (4)
C1—C20—N4—C16	−85.0 (4)	O2—Li2—Li4—O4	72.7 (4)
C19—C20—N4—O4	179.4 (4)	O3—Li2—Li4—O4	−179.0 (6)
C1—C20—N4—O4	94.7 (4)	S4—Li2—Li4—O4	−44.8 (3)
C19—C20—N4—N1	81.4 (5)	S3—Li2—Li4—O4	−178.1 (5)
C1—C20—N4—N1	−3.25 (12)	Li1—Li2—Li4—O4	42.2 (3)
C5—N1—N4—C16	10.1 (4)	Li3—Li2—Li4—O4	42.1 (5)
C1—N1—N4—C16	126.2 (4)	O4—Li2—Li4—O1^ii^	105.4 (5)
O1—N1—N4—C16	−112.2 (4)	O2—Li2—Li4—O1^ii^	178.1 (4)
C5—N1—N4—C20	−107.7 (4)	O3—Li2—Li4—O1^ii^	−73.6 (4)
C1—N1—N4—C20	8.5 (3)	S4—Li2—Li4—O1^ii^	60.6 (4)
O1—N1—N4—C20	130.1 (4)	S3—Li2—Li4—O1^ii^	−72.7 (4)
C5—N1—N4—O4	130.9 (4)	Li1—Li2—Li4—O1^ii^	147.6 (4)
C1—N1—N4—O4	−112.9 (4)	Li3—Li2—Li4—O1^ii^	147.5 (3)
O1—N1—N4—O4	8.7 (2)	O4—Li2—Li4—Li3^ii^	136.1 (4)
C5—N1—O1—Li3	−28.9 (8)	O2—Li2—Li4—Li3^ii^	−151.2 (3)
C1—N1—O1—Li3	151.2 (5)	O3—Li2—Li4—Li3^ii^	−42.8 (3)
N4—N1—O1—Li3	81.3 (5)	S4—Li2—Li4—Li3^ii^	91.3 (3)
C5—N1—O1—Li1	−133.7 (4)	S3—Li2—Li4—Li3^ii^	−42.0 (3)
C1—N1—O1—Li1	46.4 (5)	Li1—Li2—Li4—Li3^ii^	178.3 (3)
N4—N1—O1—Li1	−23.5 (3)	Li3—Li2—Li4—Li3^ii^	178.2 (4)
C5—N1—O1—Li4^i^	88.3 (5)	O4—Li2—Li4—Li1	−42.2 (3)
C1—N1—O1—Li4^i^	−91.6 (5)	O2—Li2—Li4—Li1	30.5 (3)
N4—N1—O1—Li4^i^	−161.4 (3)	O3—Li2—Li4—Li1	138.9 (4)
O2—Li1—O1—N1	138.3 (3)	S4—Li2—Li4—Li1	−87.0 (3)
O4—Li1—O1—N1	43.1 (5)	S3—Li2—Li4—Li1	139.7 (3)
S2—Li1—O1—N1	−152.7 (4)	Li3—Li2—Li4—Li1	−0.1 (3)
S1—Li1—O1—N1	−46.0 (3)	O2—Li1—Li4—O5	134.1 (5)
Li3—Li1—O1—N1	137.1 (4)	O4—Li1—Li4—O5	−128.8 (7)
Li2—Li1—O1—N1	93.0 (3)	O1—Li1—Li4—O5	−135.7 (7)
Li4—Li1—O1—N1	47.0 (6)	S2—Li1—Li4—O5	58.7 (4)
O2—Li1—O1—Li3	1.1 (4)	S1—Li1—Li4—O5	−53.7 (4)
O4—Li1—O1—Li3	−94.0 (5)	Li3—Li1—Li4—O5	163.8 (4)
S2—Li1—O1—Li3	70.2 (5)	Li2—Li1—Li4—O5	163.7 (5)
S1—Li1—O1—Li3	176.9 (3)	O2—Li1—Li4—O3	1.1 (3)
Li2—Li1—O1—Li3	−44.2 (4)	O4—Li1—Li4—O3	98.2 (5)
Li4—Li1—O1—Li3	−90.1 (6)	O1—Li1—Li4—O3	91.4 (6)
O2—Li1—O1—Li4^i^	−82.8 (4)	S2—Li1—Li4—O3	−74.3 (4)
O4—Li1—O1—Li4^i^	−178.0 (3)	S1—Li1—Li4—O3	173.3 (4)
S2—Li1—O1—Li4^i^	−13.8 (7)	Li3—Li1—Li4—O3	30.9 (5)
S1—Li1—O1—Li4^i^	92.9 (4)	Li2—Li1—Li4—O3	30.8 (3)
Li3—Li1—O1—Li4^i^	−84.0 (4)	O2—Li1—Li4—O4	−97.1 (5)
Li2—Li1—O1—Li4^i^	−128.1 (4)	O1—Li1—Li4—O4	−6.8 (5)
Li4—Li1—O1—Li4^i^	−174.1 (6)	S2—Li1—Li4—O4	−172.5 (6)
C6—N2—O2—Li3	124.1 (5)	S1—Li1—Li4—O4	75.1 (4)
C10—N2—O2—Li3	−55.7 (6)	Li3—Li1—Li4—O4	−67.3 (5)
N3—N2—O2—Li3	−161.2 (3)	Li2—Li1—Li4—O4	−67.4 (4)
C6—N2—O2—Li1	13.8 (6)	O2—Li1—Li4—O1^ii^	−100.3 (7)
C10—N2—O2—Li1	−166.0 (4)	O4—Li1—Li4—O1^ii^	−3.3 (5)
N3—N2—O2—Li1	88.5 (4)	O1—Li1—Li4—O1^ii^	−10.1 (9)
C6—N2—O2—Li2	−88.7 (5)	S2—Li1—Li4—O1^ii^	−175.8 (7)
C10—N2—O2—Li2	91.4 (5)	S1—Li1—Li4—O1^ii^	71.9 (7)
N3—N2—O2—Li2	−14.1 (3)	Li3—Li1—Li4—O1^ii^	−70.6 (8)
O4—Li1—O2—N2	−118.1 (4)	Li2—Li1—Li4—O1^ii^	−70.7 (7)
O1—Li1—O2—N2	124.2 (3)	O2—Li1—Li4—Li2	−29.6 (3)
S2—Li1—O2—N2	−12.5 (4)	O4—Li1—Li4—Li2	67.4 (4)
S1—Li1—O2—N2	113.1 (10)	O1—Li1—Li4—Li2	60.6 (6)
Li3—Li1—O2—N2	125.3 (5)	S2—Li1—Li4—Li2	−105.1 (3)
Li2—Li1—O2—N2	−118.7 (4)	S1—Li1—Li4—Li2	142.6 (3)
Li4—Li1—O2—N2	−88.0 (4)	Li3—Li1—Li4—Li2	0.1 (4)
O4—Li1—O2—Li3	116.6 (3)	O2—Li1—Li4—Li3^ii^	−32.0 (5)
O1—Li1—O2—Li3	−1.1 (4)	O4—Li1—Li4—Li3^ii^	65.1 (5)
S2—Li1—O2—Li3	−137.8 (3)	O1—Li1—Li4—Li3^ii^	58.2 (8)
S1—Li1—O2—Li3	−12.2 (11)	S2—Li1—Li4—Li3^ii^	−107.5 (4)
Li2—Li1—O2—Li3	116.0 (4)	S1—Li1—Li4—Li3^ii^	140.2 (4)
Li4—Li1—O2—Li3	146.7 (3)	Li3—Li1—Li4—Li3^ii^	−2.3 (6)
O4—Li1—O2—Li2	0.6 (4)	Li2—Li1—Li4—Li3^ii^	−2.4 (4)
O1—Li1—O2—Li2	−117.1 (3)	N1—C1—S1—Li1	−30.4 (4)
S2—Li1—O2—Li2	106.2 (2)	C2—C1—S1—Li1	147.1 (4)
S1—Li1—O2—Li2	−128.2 (10)	C20—C1—S1—Li1	73.35 (19)
Li3—Li1—O2—Li2	−116.0 (4)	O2—Li1—S1—C1	49.8 (10)
Li4—Li1—O2—Li2	30.7 (3)	O4—Li1—S1—C1	−78.6 (3)
O4—Li2—O2—N2	125.1 (3)	O1—Li1—S1—C1	38.0 (2)
O3—Li2—O2—N2	35.2 (5)	S2—Li1—S1—C1	169.1 (3)
S4—Li2—O2—N2	−158.0 (3)	Li3—Li1—S1—C1	40.4 (3)
S3—Li2—O2—N2	−37.8 (4)	Li2—Li1—S1—C1	−58.7 (5)
Li4—Li2—O2—N2	82.5 (4)	Li4—Li1—S1—C1	−108.0 (3)
Li1—Li2—O2—N2	125.7 (4)	N2—C6—S2—Li1	−5.1 (4)
Li3—Li2—O2—N2	−148.4 (5)	C7—C6—S2—Li1	172.4 (5)
O4—Li2—O2—Li3	−86.5 (4)	C15—C6—S2—Li1	−105.5 (2)
O3—Li2—O2—Li3	−176.4 (3)	O2—Li1—S2—C6	8.3 (3)
S4—Li2—O2—Li3	−9.6 (5)	O4—Li1—S2—C6	99.9 (3)
S3—Li2—O2—Li3	110.7 (4)	O1—Li1—S2—C6	−65.5 (5)
Li4—Li2—O2—Li3	−129.1 (4)	S1—Li1—S2—C6	−152.8 (3)
Li1—Li2—O2—Li3	−85.8 (4)	Li3—Li1—S2—C6	−22.2 (3)
O4—Li2—O2—Li1	−0.7 (4)	Li2—Li1—S2—C6	54.8 (3)
O3—Li2—O2—Li1	−90.6 (4)	Li4—Li1—S2—C6	103.8 (2)
S4—Li2—O2—Li1	76.3 (3)	N3—C11—S3—Li2	29.8 (4)
S3—Li2—O2—Li1	−163.5 (3)	C12—C11—S3—Li2	−147.8 (4)
Li4—Li2—O2—Li1	−43.3 (4)	C10—C11—S3—Li2	−74.09 (19)
Li3—Li2—O2—Li1	85.8 (4)	O4—Li2—S3—C11	−42.1 (11)
C15—N3—O3—Li4	27.9 (8)	O2—Li2—S3—C11	79.6 (3)
C11—N3—O3—Li4	−150.5 (6)	O3—Li2—S3—C11	−37.7 (2)
N2—N3—O3—Li4	−80.8 (6)	S4—Li2—S3—C11	−168.3 (3)
C15—N3—O3—Li2	132.2 (4)	Li4—Li2—S3—C11	−38.3 (3)
C11—N3—O3—Li2	−46.2 (5)	Li1—Li2—S3—C11	60.9 (5)
N2—N3—O3—Li2	23.6 (3)	Li3—Li2—S3—C11	109.8 (2)
C15—N3—O3—Li3^ii^	−88.3 (5)	N4—C16—S4—Li2	7.4 (4)
C11—N3—O3—Li3^ii^	93.3 (5)	C17—C16—S4—Li2	−171.9 (5)
N2—N3—O3—Li3^ii^	163.0 (3)	C5—C16—S4—Li2	108.0 (2)
O4—Li2—O3—N3	−135.9 (3)	O4—Li2—S4—C16	−10.4 (3)
O2—Li2—O3—N3	−42.6 (5)	O2—Li2—S4—C16	−100.4 (3)
S4—Li2—O3—N3	155.1 (4)	O3—Li2—S4—C16	63.4 (5)
S3—Li2—O3—N3	45.7 (3)	S3—Li2—S4—C16	152.9 (3)
Li4—Li2—O3—N3	−135.1 (4)	Li4—Li2—S4—C16	20.5 (3)
Li1—Li2—O3—N3	−92.4 (3)	Li1—Li2—S4—C16	−55.9 (2)
Li3—Li2—O3—N3	−46.2 (6)	Li3—Li2—S4—C16	−105.4 (2)
O4—Li2—O3—Li4	−0.7 (4)		

Symmetry codes: (i) *x*, *y*−1, *z*; (ii) *x*, *y*+1, *z*.

### Hydrogen-bond geometry (Å, °)

**Table:**

*D*—H···*A*	*D*—H	H···*A*	*D*···*A*	*D*—H···*A*
O5—H5A···S1^ii^	0.82	2.39	3.205 (3)	174
O6—H6A···S3^i^	0.82	2.41	3.226 (4)	172

Symmetry codes: (ii) *x*, *y*+1, *z*; (i) *x*, *y*−1, *z*.
